# The Shamiri group intervention for adolescent anxiety and depression: study protocol for a randomized controlled trial of a lay-provider-delivered, school-based intervention in Kenya

**DOI:** 10.1186/s13063-020-04732-1

**Published:** 2020-11-23

**Authors:** Tom L. Osborn, Katherine E. Venturo-Conerly, Akash R. Wasil, Micaela Rodriguez, Elizabeth Roe, Rediet Alemu, Susana Arango G., Jenny Gan, Christine Wasanga, Jessica L. Schleider, John R. Weisz

**Affiliations:** 1 Shamiri Institute, Nairobi, Kenya; 2 Department of Psychology, Harvard University, Cambridge, MA USA; 3 Department of Psychology, University of Pennsylvania, Philadelphia, PA USA; 4 Department of Psychology, Kenyatta University, Nairobi, Kenya; 5 Department of Psychology, Stony Brook University, Stony Brook, NY USA

**Keywords:** Adolescents, Sub-Saharan Africa, Global mental health, Depression, Anxiety, Shamiri

## Abstract

**Background:**

Developing low-cost, socio-culturally appropriate, and scalable interventions for youth depression and anxiety symptoms in low-income regions such as countries in sub-Saharan Africa is a global mental health priority. We developed and intend to evaluate one such intervention for adolescent depression and anxiety in Kenya. The intervention, named Shamiri (a Swahili word for “thrive”), draws upon evidence-based components of brief interventions that involve nonclinical principles rather than treatment of psychopathology (e.g., growth mindset, gratitude, and virtues).

**Methods:**

Four hundred twenty Kenyan adolescents (ages 13–18) with clinically elevated depression and/or anxiety symptoms will be randomized to either the 4-week Shamiri group intervention or a group study-skills control intervention of equal duration and dosage. Participating adolescents will meet in groups of 8–15, led by a high-school graduate trained to deliver Shamiri as a lay-provider. Adolescents will self-report primary outcome measures (depression—measured by the PHQ-8, and anxiety symptoms—measured by the GAD-7) and secondary outcome measures (perceived social support, perceived academic control, self-reported optimism and happiness, loneliness, and academic grades) at the 2-week intervention midpoint, 4-week post-intervention endpoint, and 2-week post-intervention follow-up. We predict that adolescents in the Shamiri group, when compared to the study-skills control group, will show greater improvements in primary outcomes and secondary outcomes.

**Discussion:**

Results may suggest that a brief, lay-provider delivered, school-based intervention may reduce depression and anxiety symptoms, improving academic outcomes and other psychosocial outcomes in adolescents with clinically-elevated symptoms in sub-Saharan Africa.

**Trial registration:**

Pan African Clinical Trials Registry PACTR201906525818462. Registered on 12 June 2019.

## Background

Developing low-cost and scalable interventions for youth depression and anxiety in low-income regions such as countries in sub-Saharan Africa is presently a critical priority of global mental health research [1]. The need for such interventions is warranted because while the incidence of youth depression and anxiety is on the rise, less than half of youths with depression and anxiety symptoms receive needed care [2]. In low-income regions, the burden of youth depression and anxiety is further compounded by limited treatment options [3], social stigma that prevents help-seeking [4], and government under-investment in mental healthcare resources [3, 5]. In addition, the socioeconomic stress associated with poverty (i.e., exposure to violence, inadequate access to basic needs) makes individuals in low-income communities more susceptible to developing psychological disorders [6]. As a majority of psychological disorders begin during adolescence and influence future life outcomes [3], the need for accessible interventions for youths in sub-Saharan Africa cannot be overstated. Here, we detail a protocol for a randomized controlled trial of one such intervention with high-symptom youths in Kenya.

The limited attempts at developing interventions for youth depression and anxiety in sub-Saharan Africa have focused on high-risk youths and have been derived fully or partially from formal psychotherapy interventions for mental disorders [7]. These previous interventions have included elements of cognitive behavioral therapy [8, 9] or adaptations of Western protocols such as the interpersonal therapy for adolescents (IPT-A) [10]. This approach to developing youth mental health interventions in low-income regions poses two major limitations: (1) proper delivery of interventions grounded on traditional cognitive-behavioral therapy procedures necessitates a need for rigorous training of lay-providers, which poses a barrier to dissemination and scalability, and (2) formal therapy procedures make reference to depression and anxiety, which limits help-seeking because of cultural stigma. Given the stigma associated with mental health problems in low-income countries [4, 11, 12], interventions that use non-pathologizing and non-stigmatizing language may be especially useful. As such, interventions that can circumvent both rigorous provider training and cultural stigma might be particularly useful. One such intervention is the Shamiri (“thrive” in Swahili) group intervention for youth depression and anxiety that is delivered by lay-providers in school settings [13].

The Shamiri intervention draws upon evidence-based components of brief scalable interventions that involve non-clinical psychological principles (rather than treatment of psychopathology). Specifically, Shamiri draws on previous research on brief single-component interventions that are sometimes called “wise interventions” [14, 15], some of which have been found to improve youth mental health outcomes [16]. Wise interventions (WIs) are brief and require minimal training of lay-providers. Furthermore, they emphasize overall human functioning rather than referring to mental health disorders. Thus, wise interventions may circumvent social stigma in a way that formal psychotherapy techniques do not [16]. The specific WI components of the Shamiri protocol are growth-mindset [17–19], gratitude [20], and value affirmations [21]. The Shamiri intervention has been trialed previously with Kenyan youths. In a randomized controlled trial of the group-based Shamiri with high-symptom youths in an urban slum (*N* = 51, ages 14–17), adolescents who met a clinical cut-off for depression and anxiety were randomized into either the Shamiri intervention or an active study-skills control group; those assigned to the intervention group showed improvements in youth depression and anxiety symptoms, academic performance, and perceived social support from friends [13]. However, the trial was limited by a modest sample size and limited follow-up. Consequently, a replication of the Shamiri intervention with larger sample size is a logical next step.

This study has two primary objectives. The first is to evaluate an enhanced Shamiri group intervention—which incorporates student and stakeholder feedback from the previous Shamiri trial [13]—for depression and anxiety for youths with elevated symptoms in Kenya. The Shamiri intervention aims to be low-cost, to be positively focused in order to circumvent stigma, and will rely on empirically supported principles that can be delivered in a low-resource community setting by lay-providers. The second objective is to conduct moderator and mediator analyses on study outcomes to identify for whom and under what circumstances the treatment works.

The present study’s primary outcomes are youth depressive and anxiety symptoms. The secondary outcomes are social support, perceived control, gratitude, loneliness, emotional regulation, and academic outcomes. We predict that youths assigned to the Shamiri intervention will experience reductions in primary outcome measures and improvements in secondary outcomes measures when compared to youths assigned to an active “study-skills” control group from baseline to post-intervention endpoint. We opted for an active study-skills control group because we wanted all participants to have an opportunity to benefit from participation [13] and because a recent meta-analysis of youth psychotherapy indicated that active control conditions provide a more rigorous standard of comparison as opposed to passive controls such as a waitlist [22]. Including a control condition comparable in dose and intensity to the treatment condition also helped to ensure that any condition difference in study outcomes could be attributable to intervention content rather than differences in dose or intensity of the two study conditions.

## Methods

### Study design

#### Trial design

The trial is a randomized controlled trial with two arms: the Shamiri intervention and a study-skills control intervention. The intervention and control conditions are described in detail later and summarized in Table 1. Interventions will be delivered in schools to groups of 10–13 youths by high school graduates trained to be lay-providers. Assessments will be administered at initial screening, 2-week midpoint, 4-week endpoint, and 2-weeks post endpoint. Academic performance data on grades and national exams will be collected for the school-term before, during, and after study participation. Any data required to support the protocol will be publicly available in an online repository [10.17605/OSF.IO/KTW25] or upon request.

**Table 1:** Arms of the Shamiri randomized control trial

Study arm	Content	Delivery
Shamiri intervention	Consists of four sessions made up of three modules: growth-mindset (two sessions), gratitude (one session), and virtues (one session).	High school graduates trained as lay-provider (“group leader”). One group leader will be assigned to each group of 10–13 youths.
Study-skills control	Consists of four sessions with content that focus on study skills. Specific modules will include note-taking, effective study strategies, tips for time management, and the study cycle. Equal dose and duration as Shamiri intervention.	High school graduates trained as lay-providers (“group leader”). One group leader will be assigned to each group of 10–13 youths.

#### Study setting

This study will take place in four secondary schools located in Nairobi and neighboring Kiambu Counties, Kenya. Nairobi County is one of 47 geographical counties that act as units of devolved government in Kenya, a low-income country in sub-Saharan Africa. Nairobi is a diverse city with a population of at least 3.2 million that is representative of the cultural and socioeconomic diversity in Kenya [23]. Kiambu County, a county that is part of the former Central Province region, neighbors Nairobi County and has a population of at least 1.7 million [23]. The first author will draw a list of public secondary schools in Nairobi and Kiambu counties. Principals of the schools will be contacted with information about the study and asked to enlist their school to participate. From the pool of interested schools, four schools that have strong demographic, socioeconomic, and educational diversity will be selected. In particular, the selection will be done in such a manner that schools that are ranked as national secondary schools (which have a socio-economically diverse student body from all the regions in Kenya) and sub-county secondary schools (which are often resource-poor day single-sex or mixed secondary schools with student body from the local area) by the Ministry of Education, Science and Technology will be selected—more information on the diversity of secondary schools in Nairobi and Kiambu counties can be seen elsewhere [24, 25]. In total, the final school sample will consist of an all-boys *national* boarding secondary school, an all-girls *national* boarding secondary school, an all-girls *sub-county* day secondary school, and a *sub-county* mixed-day secondary school. This diverse and generalizable sample of high-symptom youth in Kenya will also allow us to conduct robust moderator analyses that can allow us to identify for whom the Shamiri intervention works for.

As reported elsewhere [13], the Shamiri intervention was designed through close multicultural collaboration by experts from Kenyan and American institutions (the first author, for example, is a Kenyan citizen who went through the Kenyan public education system and brings firsthand experience of the same). Kenyan stakeholders (school administrators, students, and community members) were also extensively involved in the intervention development and adaptation process to ensure that the intervention was socio-culturally appropriate for the Kenyan context [13]. Such multicultural collaboration is particularly necessary for global mental health research [26].

### Procedures

#### Participant recruitment and screening

In all four participating schools, students in Form One, Form Two, and Form Three (and in smaller schools Form Four) will be notified about the study by the school principals and other administrators. All students aged between 13 and 18, inclusive, will be eligible for the study if they self-report either elevated depression symptoms or elevated anxiety symptoms—indexed by a score of 15 or higher on the Patient Health Questionnaire (PHQ)-8 (indicating moderate or severe depression) or a score of 10 or greater on the GAD-7 (indicating moderate or severe anxiety) [27, 28]. No other exclusion criteria will be applied. Of eligible students, approximately 140–180 will be randomly selected at each school to participate in the study. See the “Randomization, treatment allocation, and blinding” section for more information on selection and randomization.

**Fig. 1:**
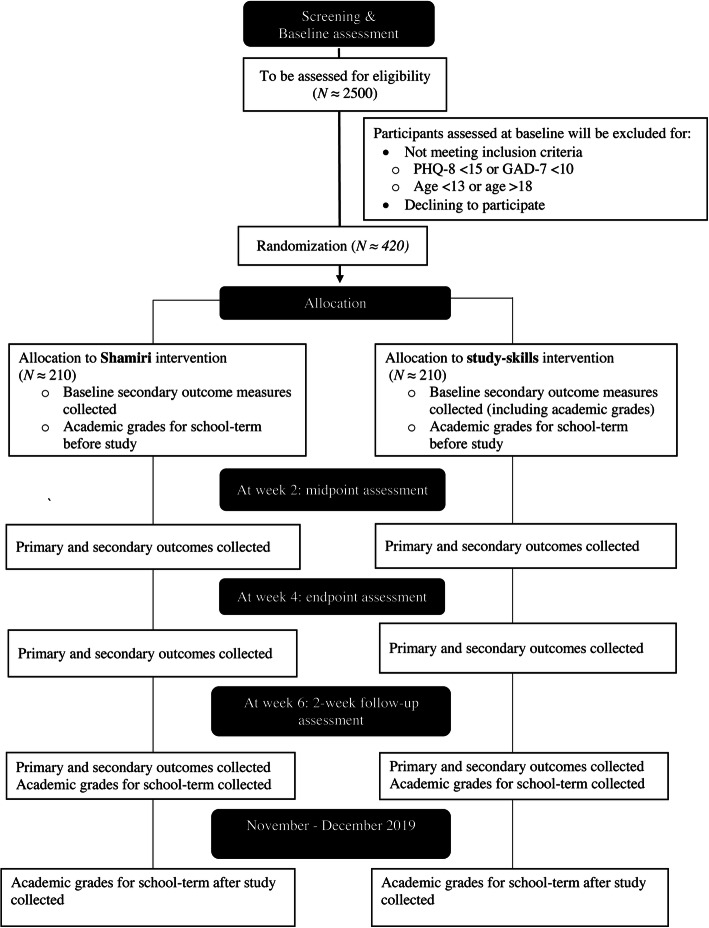
CONSORT flow diagram

Students will complete a questionnaire battery to determine study eligibility. To control for ordering effects, scales in the questionnaires will be presented in different orders. Parental consent will be sought for all minor participants. As the schools in which we will be conducting study activities are boarding schools, many of the students will not have direct access to their parents [24, 25]. Additionally, because of the logistical difficulties of the study team obtaining written consent from parents who live in many different parts of the country—many quite distant from the schools and not readily accessible—the Ethics Review Committee at MUERC determined that school principals and other administrators would seek parental/guardian permission from parents of the students interested in the study using their locally available policies and resources—for example, a school in Nairobi county with students from all the geographical counties in Kenya may call or write parents/guardians—per MUERC guidelines. This process was used in the pilot study [13] and has been used in multiple studies with Kenyan adolescents [24, 25, 29]. Only students for which parental permission will be obtained through this process will be allowed to participate in the study. School administrators will then gather these students in classrooms or school halls where they will meet the study team. Here, the study team will further inform them about the study and that their participation is voluntary and that they can opt out. After being provided with an opportunity to ask questions, they will provide informed consent if they wish to participate. Only participants for whom informed consent or informed assent and parental consent (in the case adolescent minors) is received will be allowed to participate. The study team and a school administrator will witness the process of obtaining informed consent. On the consent form, participants will be asked if they agree to the use of their data should they choose to withdraw from the trial. Participants will also be asked for permission for the research team to share relevant data with people from the Universities taking part in the research or from regulatory authorities, where relevant. This trial does not involve collecting biological specimens for storage. There is no anticipated harm for trial participation and there is no provision for post-trial care. See Fig. 1 for a participant flowchart.

#### Randomization, treatment allocation, and blinding

Following baseline assessment and recruitment, youths who meet the inclusion criteria will be randomly allocated within each school to the Shamiri intervention or the study-skills control condition using a computerized random number generator in R Studio. We will use stratified randomization to ensure consistent baseline characteristics (age, form, and sex if mixed school) across groups. There will be no special criteria for discontinuing or modifying allocated interventions. Participants and group leaders will not be blinded to treatment allocation. However, they will not be informed of study hypotheses. To control for possible contamination, the study will be presented to all eligible students as a Wellness and Academic Functioning Program that was intended to improve both academic functioning and wellness. Both conditions will also be of equal duration and will require similar efforts, and group leaders will be instructed to collect study materials at the end of each session (to prevent students from noticing differences in their program materials and learning from others’ materials during the study).

#### Group leader selection and training

Both the Shamiri and study-skills groups will be led by trained group leaders who will serve as lay-providers. To qualify as a group leader, an individual must be a Kenyan high school graduate between the ages of 17 and 26, inclusive, and he or she must live in the local community in and around Nairobi. While most group leaders will be either planning to matriculate or already attending a local university in Nairobi, neither college admittance nor attendance is a requirement for consideration. The above group leader characteristics are important for the successful implementation of this protocol for several reasons: (1) Local group leaders’ understanding of the culture, language, and local customs in schools make them better-positioned to deliver the intervention than outsiders. (2) Group leaders who are closer in age to the youths in the program may be better positioned than older adults to connect with youths—this is because Kenyan cultural norms around age relations dictate that youths should primarily listen rather than speak in the company of adults. (3) Lastly, this model is potentially scalable: given that the mean age in Kenya is 19, there is a large pool of youths aged 17–25 from which group leaders could be selected—median age is 19 [30].

We will recruit group leaders through advertisements in high school graduate platforms on various media such as WhatsApp and Facebook. We will also recruit through local universities and organizations in Kenya that work with high school graduates. All interested group leaders will be offered an opportunity to express their interest via a brief online form. Group leaders who meet the above-specified criteria will be invited for a 25-min interview with members of the study team. This interview will assess past teaching experiences, interest in the project, familiarity with mental health and wellness issues, and interpersonal facilitation skills. Chosen group leaders will then be contacted via email and phone and invited to a mandatory 10-h training. Due to funding constraints, we intend to recruit 12 group leaders. All group leaders will be compensated $150 plus the cost of transportation for the entire duration of the study.

The group leader training, which will be conducted by members of the research team, will begin with 4 h of general communication and group leadership skills such as active listening, noting connections between group members, handling conflicts within the groups, and referring students in need to appropriate school officials if necessary. Group leaders will then be trained didactically in the specific content of each week of the interventions. All group leaders will receive detailed and structured outlines of the content of each intervention and control session. The outlines will include sample wording for the group leaders. The last six of the 10 h of training will consist of role-plays in which the leaders practice leading groups in portions of the protocol. Following the role-plays, leaders receive feedback from their fellow group leaders and from the study team. The leaders are then asked in the full group setting to reflect on their experiences and learnings from the role-plays. A detailed protocol for the recruitment and training of group leaders is available elsewhere [30].

Upon completion of training, group leaders will be randomly assigned to lead either Shamiri intervention or control groups. Given that we will have about 48 groups of up to 15 youths in the entire study, group leaders will be randomly assigned to either the intervention or control condition at each school. That is, the randomization process will be repeated for each distinct school. For instance, one group leader might lead a study-skills control group in School A but a Shamiri intervention group in School B. This randomization should allow us to account for group leader differences in competence and leadership style. However, group leaders will be instructed to strictly follow the protocol manual for the condition that they will be leading and not use content from the other condition during their sessions. Student composition in the groups will reflect the composition of the school. In a single-sex school, all groups will be single-sex.

### Intervention arms

#### Shamiri intervention

The Shamiri intervention consists of three modules: a growth-mindset module [17, 19, 31] lasting two sessions, a gratitude module [20] lasting one session, and a virtues module based on value affirmations [21, 32] lasting one session. More detailed information on these sessions is provided below.

##### Growth-mindset interventions

Growth-mindset interventions target implicit theories about the ability of human attributes to evolve over time. As such, growth-mindset interventions challenge the belief that personality traits and intelligence are fixed and unchangeable. These interventions are designed to strengthen individuals’ beliefs in the malleability of such characteristics [19, 33]. Meta-analyses of growth mindset interventions have shown robust improvements in academic achievement, particularly among low-income students [34].

##### Gratitude interventions

Research on trait gratitude suggests that grateful people exhibit more positive states and outcomes than non-grateful people. Gratitude interventions teach individuals to be more aware of and thankful for the positive people and things in their lives, regardless of whether they are tangible or not [20].

##### Value affirmations interventions

Value affirmation interventions invite people to reflect on specific values that are most important to them. This self-reflection can improve people’s coping skills by reestablishing an awareness of personal worth and integrity [21, 32]. Due to differences in language and vocabulary, the discussion of values will be presented to Kenyan youths as “virtues.”

#### Structure of intervention sessions

The intervention consists of four intervention sessions, which are each 1-h long and 1 week apart. All of the sessions include reading activities, group discussions, and writing activities. Homework exercises are assigned between sessions. In sessions one and two, participants learn about growth, neuroplasticity, and work to build their growth-mindsets. In sessions three and four, participants learn about gratitude and value affirmations respectively.

##### Session one: growth

In session one, the group leader begins with group introductions and an ice-breaker activity. Then, the group leader provides a didactic introduction to growth and personal improvement in different areas—social, academic, and emotional—and their benefits. Participants then read an article and watch a short video that explains neuroplasticity: a phenomenon whereby the brain grows and makes new connections as a result of learning and practicing new skills. Both the article and video script are available in supplementary materials; the video is available upon request and online. Next, the participants read three growth testimonials from local Kenyan peers and a growth testimonial from their own group leader (see supplementary materials). Testimonials focus on personal growth in a plethora of domains (e.g., school performance, personality, happiness, relationships). Participants then have a discussion about the testimonials. To conclude the first session, participants are assigned a take-home activity in which they identify a challenge they faced, describe how they used effortful strategies to deal with the challenge, and reflect on how they grew as a result of the challenge.

##### Session two: growth

In session two, participants begin by discussing their take-home activity. They then brainstorm and discuss effective strategies that they can use to apply the lessons of growth-mindset in their own lives. Next, the group leader will moderate a discussion about problem-solving skills modeled off of the problem-solving STEPS module [35]. Afterward, participants are instructed to write a letter to a friend in which they explain the concepts that they have learned thus far—including neuroplasticity, growth-mindset, effective strategies, and problem-solving skills—in their own words. The group leader is instructed to do all of the same activities themselves while the participants do them. Lastly, participants are assigned a take-home activity in which they think of one problem that currently affects them and brainstorm solutions modeled off the problem-solving skills learned in the session. They are encouraged to try one or more of these solutions during the week.

##### Session three: gratitude

In session three, students learn about gratitude. The group leader opens the session with a didactic introduction to gratitude and its various benefits. The participants then discuss gratitude and identify people and things for which they are grateful. After this, participants write a “gratitude letter” [36] to a living person who has changed their life for the better. For homework, participants are instructed to complete a daily “three good things” activity during the following week [37]. In this activity, participants identify and write three good things that happened each day and write a short reflection for each.

##### Session four: virtues

In session four, students learn about virtues and their importance in everyday life. While the activities in this section are based on empirically-tested value affirmations exercises, the discussion of values will be presented to Kenyan students as “virtues” due to differences in language and vocabulary. The group leader opens the session with a didactic introduction to what virtues are. The group then enters a discussion about virtues, noting why they are important in guiding daily behavior and helping us find purpose in our lives. Students are then asked to select from a long list several virtues that are meaningful to them. Next, students select the one particular virtue that they feel is most important to them and describe, in writing, why this virtue is important, a time when they have lived up to that virtue, and how they can live in better accord with this virtue in the future [21]. There is no take-home activity following this fourth and last session. At this point, participants are allowed to keep the Shamiri booklet, which contains all the lessons and exercises from the four sessions for both the Shamiri intervention and the control. Session four also last for 40 min to allow students ample time to complete endpoint assessment.

#### Study-skills control

Using a study skills control group both presents participants with an opportunity to benefit from participation and provides a more rigorous standard of comparison than passive controls [22].

The control condition consists of four modules: a note-taking module lasting one session, an effective-study-strategies module lasting one session, a time management module lasting one session, and a study cycle module lasting one session. All these modules were developed specifically for a pilot trial of the Shamiri intervention [13]. More information on these sessions is provided below:

- *Note-taking*: Developing effective note-taking skills may help students improve their academic performance. The session will cover what the study team will present as the 5Rs of note-taking: Record, Reduce, Recite, Reflect, and Review (see online supplementary materials for more information).
- *Effective study strategies*: Effective study strategies may help students understand what they are learning and how they can optimize their time spent studying. The session will cover 10 effective study strategies compiled by the study team, including “have a study plan,” “don’t cram all studying into one session,” and “start with the most difficult subject” (see online supplementary materials for more information).
- *Time management*: Time management skills may help people become more productive and improve academic performance. This session will cover four steps that may help people manage time more effectively: organizing time, assessing time, setting priorities, and scheduling time (see online supplementary materials for more information).
- *The study cycle*: The study cycle is a 5-step approach to learning that may help students’ academic performances by reinforcing new content and making them feel more confident. This session will cover the 5 steps of the study cycle: preview, attend, review, study, and assess (see online supplementary materials for more information).

#### Structure of the control sessions

To control for non-specific aspects of the intervention, the study skills control group (developed for the present study) will mirror the structure of the Shamiri intervention. Each condition contains the same number of exercises and group discussions, within-session activities are similar in format (e.g., reading, writing, and discussion activities), and between-session take-home assignments require similar effort.

As detailed, the study skills control consists of four sessions, which are each one-hour long and 1 week apart. Take-home assignments are also assigned between sessions.

##### Session one: note-taking

During session one, group leaders will offer a didactic introduction to the 5Rs of note-taking. Then, participants will complete an exercise to practice the new note-taking method: They will read a short article about climate change and take notes using the 5Rs strategy they just learned. For homework, participants will be asked to complete three tasks: they will (1) identify one particular class during which they used the 5Rs of note-taking, (2) describe how they used the 5Rs to take notes, and (3) reflect on whether their learning improved as a result of using the new note-taking strategy.

##### Session two: effective study strategies

To begin session two, participants will reflect on the homework in a group discussion (e.g., What was rewarding or challenging about using the 5Rs? Do you plan to use them in the future?). Then, group leaders will introduce ten effective study strategies. Participants will be asked to discuss these ten strategies, examples of when they have used them, and other strategies that have worked for them. For homework, participants will be asked to complete three tasks: they will (1) identify a specific academic challenge they face over the course of the following week, (2) employ one strategy from the ten discussed in this session to address the challenge, and (3) reflect on whether their learning improved as a result of using the new strategy.

##### Session three: time management

To begin session three, participants will reflect on their homework assignment in a group discussion (e.g., Which strategies worked for you? Which ones did not? Why? What strategies might you use in the future?). Then, group leaders will introduce the concept of time management and lead a discussion about the four steps to improving it as a skill. For homework, participants will be asked to track and write down how they spend their time on activities like schoolwork, sleeping, and eating. This homework is intended to help participants understand how they are actually spending their time each day.

##### Session four: the study cycle

To begin session four, participants will reflect on their homework assignment in a group discussion. Group leaders will ask if anybody is willing to share their time management worksheet and discuss with the group. Then, group leaders will introduce the five-step study cycle and discuss it with the students. Finally, group leaders will lead a discussion about how participants can continue practicing skills they have learned from all the sessions in the future. There is no take-home activity following this fourth and last session. At this point, participants are allowed to keep the Shamiri booklet, which contains all the lessons and exercises from the four sessions of the study-skills sessions. Session four also lasted for 40 min to allow students ample time to complete endpoint assessment.

#### Intervention fidelity

We will assess intervention fidelity—including group leader adherence to protocol manual and group leader competence in delivering the Shamiri and study-skills interventions—using a fidelity rubric (see Table 2) developed by the study team. In this rubric, sessions are broken down into small 5–10-min chunks that reflect the activities which are outlined in the protocol manual. Raters will listen to and rate these chunks. They will code group leader adherence to protocol manual (i.e., whether a group a leader followed manual and delivered required content classified as 0 = no, 1 = yes) and group leader competence (i.e., effectiveness in communicating concepts and skillfulness of delivery, rated from 1 = not-competent to 5 = very competent). Ten percent of the total sessions will be assessed for fidelity.

**Table 2:** Intervention fidelity rubric

The group leader followed the study protocol and delivered the required content	
The group leader adhered to the specifications around the content delivered (i.e., handed out necessary articles/materials, facilitated discussion)	
In your opinion, how thoroughly did the group leader deliver the required content	
In your opinion, how skillfully did the group leader deliver the required content	
In your opinion, did the group leader deliver the required content in a clear and accessible manner	

Raters (*N* = 2) will be recruited from psychology or social science departments at local universities in Nairobi. The raters will be blind to study purposes. They will first undergo a series of trainings led by the first author, including a didactic training on the protocol manuals of the Shamiri and study-skills groups, a training of the fidelity rubric, and training on the 5–10-min chunks. The raters will then listen to a randomly selected sample from each of the conditions, and rate them independently. After this, the raters will work with a member of the study team and walk through their ratings. Next, the raters will grade a randomly selected 10% of the audio-recordings of the Shamiri and study-skills sessions.

### Measures

#### Primary outcome measures

##### Patient Health Questionnaire-8

We will use the 8-item version of the Patient Health Questionnaire-9, the Patient Health Questionnaire-8, to assess youth depression symptoms. PHQ-8 scores are highly correlated with PHQ-9 scores, and the same cutoffs can be used to assess depression severity [27, 38]. The PHQ has previously been used with both Kenyan adolescents [25] and adults [39, 40].

##### Generalized Anxiety Disorder Screener-7

The Generalized Anxiety Disorder Screener-7 (GAD-7) [28] is used globally to screen for generalized anxiety disorder in adolescents and adults. The GAD-7 has previously been used with both Kenyan adolescents [25] and adults [39, 40].

### Secondary outcome measures

#### Academic performance

##### Average term grade

Academic grades of the participants will be collected for three school-terms: the school-term before the intervention (January to April 2019), the school-term during the intervention (May to August 2019), and the school-term after the intervention (September to November 2019). To determine students’ average term grade, we will calculate a mean term grade from their grades in all the subjects in which they will be enrolled. Although subject enrollment varies across different schools, students are typically expected to enroll in between 6 and 12 subjects. In order to compare students’ grades across different grade levels, academic subjects, and schools, we will convert the academic grades to standard scores (M = 60, SD = 10, chosen arbitrarily and used in rescaling); this standardization was piloted in our pilot RCT of this intervention [13].

##### Science and math score

We will calculate a science and math score for each of the three semesters for which academic grades will be collected. The score will be calculated as the mean grade of the math, biology, chemistry, and physics grades. As with the average term grade, we will convert these grades to standard scores with M = 60, SD = 10 used in rescaling.

##### Humanities and languages score

We will calculate a humanities and languages score for each of the three semesters for which academic grades will be collected. The score will be calculated as the mean grade of the English, Kiswahili, history, geography, and religious studies grades. As with the average term grade, we will convert these grades to standard scores with M = 60, SD = 10 used in rescaling.

##### Self-reported perceived academic performance

We will ask students to self-report on how they perceive their academic performance. This will be a one-item instrument that will ask them to self-report their perceived academic performance on a 5-point Likert scale from not satisfactory to excellent. The item has been used in a previous study with Kenyan university students [41].

##### Kenya Certificate of Primary Education (KCPE) scores

The KCPE is a national examination that all students take at the end of 8 years of primary school and determine admission to secondary school [24, 25]. KCPE scores will be collected for all participants, not as an outcome measure, but as a potential moderator of changes in academic performance outcomes.

#### Multidimensional Scale of Perceived Social Support

The Multidimensional Scale of Perceived Social Support, MSPSS [42], is designed to measure satisfaction with social support. It consists of three subscales: the “friends” subscale (which measures support from friends), the “family” subscale (which measures support from family), and the significant others subscale (which measures support from significant others). The MSPSS has previously demonstrated adequate internal consistency in Kenyan adolescents [25].

#### Perceived Control Scale for children

The Perceived Control Scale, PCS [43], includes 24 items related to beliefs about personal control, or the belief that, through effort, one can obtain desired outcomes and avoid undesired outcomes. Example items include “I can make friends with other kids if I really try” and “I cannot stay out of trouble no matter how hard I try.” The PCS has previously demonstrated adequate internal consistency among Kenyan adolescents [25]. We will use the social control and academic control subscales as secondary outcome measures.

#### The Gratitude Questionnaire

The Gratitude Questionnaire (GQ-6) is a six-item questionnaire that measures grateful disposition—the tendency to experience and act with gratitude in everyday life [44]. The GQ-6 has been shown to have strong psychometric properties among North American youths though a previous attempt to use the GQ-6 with Kenyan youths demonstrated weak internal consistency [25].

#### The EPOCH Measure of Adolescent Well-Being

The EPOCH Measure of Adolescent Well-Being assesses positive psychological characteristics that might foster well-being, physical health, and other positive outcomes in adulthood. While the EPOCH has been validated with adolescents in Australia and the USA [45], it has yet to be used with adolescents in Kenya and other sub-Saharan African countries to the best of our knowledge. We will use the Optimism and Happiness subscales of the EPOCH.

#### UCLA Loneliness Scale – shortened version

The UCLA Loneliness Scale is a widely validated tool for measuring trait loneliness. Participants rate their responses to statements such as “I feel isolated from others” and “people are around me but not with me*”* on a 4-point Likert scale. The third and newest edition of the UCLA Loneliness Scale has demonstrated high reliability, construct validity, and discriminant validity [46]. We will use a shortened version of the scale (8-item) of the UCLA Loneliness Scale, which shows similar validity and reliability to the standard 20-item scale [46]. To the best of our knowledge, it has yet to be used with adolescents in Kenya.

#### Program feedback scale

A feedback scale will be used to collect acceptability and feasibility data from participants. The youths will be asked questions about whether they found the program useful, whether they understood the contents of the program, whether they would recommend the program to a friend, which were the most and least helpful elements of the program, and how they would rate their overall experience. They will also have the option to recommend changes to improve the program for future participants. This scale was developed specifically for this study; however, several items were drawn from prior research [13, 47]. The scale is available in Table 3.

**Table 3:** Program feedback scale. Items rated on a 1 (“strongly disagree”) to 5 (“strongly agree”) scale unless otherwise specified

I enjoyed/liked participating in the program	
The program as a whole was helpful	
The material in this program was easy to understand	
The homework activities were helpful	
I liked my group leader	
My group leader was helpful	
I agree with this program’s message	
What lesson did you find most useful? If in Shamiri intervention group, options are A) growth-mindset, B) gratitude, V) virtues, and if in study-skills control group, options are A) note-taking, B) effective reading strategies, C) tips for time management, D) study cycle	
What lesson did you find least useful? If in Shamiri intervention group, options are A) growth-mindset, B) gratitude, V) virtues, and if in study-skills control group, options are A) note-taking, B) effective reading strategies, C) tips for time management, D) study cycle	
What was your favorite thing about the program? (free response)	
What do you think we can change to improve the program? (free response)	

#### Timeline for study and data collection

Participant recruitment will begin in mid-to-late June 2019. The intervention will begin in mid-to-late June 2019 and end in mid-August 2019. Data collection will end in December 2019, once final follow-up measures are collected. We intend to report results by the end of 2020. Upon termination of data collection and publication of results, we will make study data publicly accessible at this link [10.17605/OSF.IO/KTW25]. To protect participant confidentiality, we will not provide identifiable data.

### Data analysis plan

#### Sample size and statistical power

Optimal Design (OD) [48], a statistical simulation program for estimating power and sample size for multi-level models, was used to estimate sample size. Based on a pilot study of the Shamiri intervention [13] which revealed an effect size of *d* = .32 for depression and an effect size of *d* = .54 for anxiety, we calculated the sample size necessary to detect an effect of *d* = .3 using an alpha value of *p* = 0.05 and a power of 0.80, accounting for the repeated measures design with five measurement points by specifying settings on OD, and without consideration of covariates or clustering. Using these parameters, at least 200 participants are required per group for a total of at least 400 participants; we aimed to enroll 420 participants. Assuming that 30% of willing students at recruited schools will be eligible to participate in the study—based on previous assessment wherein 30% of youths in 5 participating secondary schools in Kenya showed elevated symptoms of depression and anxiety [13] and projecting that up to 10% of students may discontinue from beginning to midway through the intervention—based on a conservative estimate from the drop-out rates of Osborn et al. [13], at least 1500 youths are needed for baseline screening to realize the minimum sample size. Given the participation of 4 Kenyan secondary schools in the present study with 3200 students enrolled, this sample size is believed to be feasible [13].

#### Interim analyses and stopping guidelines

We do not anticipate problems that are detrimental to the participants during the course of the RCT and subsequent follow-up data collection; as such, we do not have stopping guidelines and do not intend to conduct interim analyses until follow-up data collection is completed.

#### Data description: nesting, missingness, and imputation

There will be four levels of clustering in our data: school (level 4), group-leader (level 3), groups (level 2), and participant (level 1) with repeated measures being the individual observations at the multiple timepoints (level 0). Group leaders and schools will be cross-classified in an unstructured manner as group leaders will be able to lead groups in multiple schools. Groups will be strictly nested within group leaders as participants will be within groups (structured cross-classification). Missing item (measurement) and subject-level data will be imputed twenty times using Fully Conditional Specification (FCS), implemented using the multivariate imputation by chained equations (mice) algorithm in R as described by Buuren and Groothuis-Oudshoorn [49].

#### Analysis of primary and secondary study outcomes

Outcome measures will need to demonstrate internal consistency with Kenyan youths as indexed by a Cronbach’s alpha of at least 0.70 [50] to be considered for further analyses.

Our analyses will follow an intent-to-treat approach, including all participants initially randomized to an intervention condition. We will run a linear mixed-effects model comparing intervention and control groups on each primary and secondary outcome. Models will be organized to reflect the hierarchical structure of the data described earlier with crossed random effects for schools and group leaders and strict hierarchical nesting for observations in participants, participants in groups, and groups in group leaders. All models will include a random intercept that will allow for individual variation at baseline. We will include a random slope that will allow for individual variation in outcome change rates; however, should this result in overfitting (i.e., random effects structure becoming too complex to be supported by the data), we will remove the random slope to allow for more parsimonious models. Time, intervention condition, and their interaction will be included in all models. Covariates will include age (in years) and sex. Age will be included because older adolescents are reported to face increased psychosocial stress, which may exacerbate depressive and anxiety symptoms [25, 51]. Sex will be included because sex differences in internalizing problems have been documented in Kenyan adolescents [24, 25, 29, 52]. We will build two models, one which will include schools a random slope thus allowing for variation in outcome change rates by school, and another with school as a covariate because students in resource-poor schools in Kenya have reported higher anxiety symptoms than students in schools with more resources [25]. We will use Akaike Information Criterion (AIC) to determine which of the two models will be a better fit for the data. Significant (*p* < .05) condition × time interactions in predicted directions will indicate that the intervention condition produced more rapid improvements in outcomes across the full study period (baseline to 4-week endpoint), as compared to the control.

We will calculate effect sizes (ESs) for total pre- to post-intervention change in depressive symptoms (the primary study outcome) using differences in means; these ESs will compare mean gain scores (Cohen’s *d*) reflecting changes in each outcome from baseline to post-treatment for youths in the Shamiri intervention versus study skills control intervention. Statistically significant, positive Cohen’s *d* values will indicate greater improvements for intervention group youths versus control group youths.

Participants who miss one or more intervention sessions will be permitted to attend future sessions. Missing data will be imputed as described earlier. As part of our main analyses, we do not plan to carry out additional analyses such as subgroup analyses to gauge for intervention effects on primary and secondary outcomes. However, we may later conduct additional exploratory analyses (e.g., moderator analyses) in papers separate from those reporting main and secondary trial outcomes.

### Quality control and trial management

The management structure comprises of the co-principal investigators (PIs), a research management group, and a data monitoring committee. The research management group is responsible for conducting the trial—including recruiting participating schools and students, obtaining consent, and other study activities—and will meet weekly to discuss trial progress. The PIs will oversee the research management group meetings. The research management group will provide day-to-day on-site support for group leaders and research assistants. The data monitoring committee—which will consist of the PIs who are affiliated with Shamiri Institute and independent members affiliated with the Jomo Kenyatta University of Agriculture and Technology and Kenyatta University—will review safety data as well as oversee the collection and entry of data from the study. Data from the paper questionnaires, which were stored in a locked room in study headquarters, will be double-entered by members of the study-team into a secure and encrypted Excel database. To ensure data quality, processes like double data entry, random data checks, and range check for data values will be adopted under the guidance and supervision of the research management team. Data will be stored in a secure database which will be locked after all the data have been cleaned and entered. During the study, the research management group will collect spontaneous and unsolicited reports of adverse events and other unintended effects of trial interventions or trial conduct. One member of the research team, who is a clinical psychologist, will serve as the point person for any information about adverse events, participant risk, or trial conduct. Upon receiving such information, the PIs will reach out to the MUERC for guidance. Trial conduct will be assessed by the MUERC. Finally, any modifications to the trial protocol (including changes to eligibility criteria, outcomes, and analyses) will be reported to the MUERC, documented in the trial registry, made public in the trial’s Open Science Framework’s repository, and circulated to all relevant parties.

### Ethical considerations

Study methods and protocols have been approved by the Maseno University Ethics Review Committee (MUERC) reference number MSU/DRPI/MUERC/00727/19.

## Discussion

In Kenya and similar countries in sub-Saharan Africa, there exists a stigma around help-seeking and a scarcity of providers and services compounds the burden of psychopathology [3–5]. Still, there is a particularly high prevalence of depression and anxiety symptoms in school-going adolescents [24, 25, 29]. This protocol details the procedures for the Shamiri intervention, a lay-provider delivered and school-based intervention for adolescent anxiety and depression. A pilot trial of the Shamiri intervention showed that the intervention reduced depression and anxiety symptoms for high-symptom adolescents [13]. A recent pilot trial of an online single-session version of Shamiri also showed that the intervention reduced depression symptoms for youths with effect sizes exceeding traditional psychotherapy [53]. Should this trial of Shamiri also show clinical utility, it may provide further support for a potentially low-cost help-seeking avenue for high-symptom Kenyan youths. Beyond potential clinical utility, should Shamiri improve academic outcomes then these effects on school performance may be especially relevant to contexts where mental health stigma works against help-seeking. Such beneficial effects on school performance will likely be valued by school personnel, students, and parents, regardless of prevailing societal attitudes toward mental health.

Shamiri appears to be cost-effective and scalable relative to traditional psychological interventions. The group-based format and use of lay-clinicians in particular enhance cost-effectiveness and scalability. The group format may increase the number of youths who have access to the intervention and using lay-clinicians may help address the shortage of mental health specialists. In addition, the positive emphasis of the Shamiri intervention may serve to reduce social stigma around depression and anxiety. Youths do not directly talk about depression and anxiety in the groups and the intervention is not presented as a depression and anxiety treatment.

This intervention might be a valuable step-forward in increasing help-seeking options that can lighten the global burden of adolescent depression and anxiety.

## Trial status

Completed. Protocol version number: 1.2; original date: 10th June 2019; updated: August 30th, 2020.

Participant recruitment start date: 18th June 2019; participant recruitment completion date: 18th July 2019.

## Supplementary Information

**Additional file 1.**

## Data Availability

Beginning 3 months and ending 3 years following article publication that result from the present trial, individual participant data that underlie the results reported in the article, after de-identification (text, tables, figures, and appendices).

## Abbreviations

IPT-A: Interpersonal therapy for adolescents
WI: Wise interventions
PHQ-8: Patient Health Questionnaire-8
GAD-7: Generalized Anxiety Disorder Screener-7
STEPS: An abbreviation for a problem-solving module
KCPE: Kenya Certificate of Primary Education
MSPSS: Multidimensional Scale of Perceived Social Support
PCS: Perceived Control Scale
GQ-6: Gratitude Questionnaire – 6
EPOCH: Engagement, Perseverance, Optimism, Connectedness, and Happiness Scale
MUERC: Maseno University Ethics Review Committee

## References

[1] Collins PY, Patel V, Joestl SS, March D, Insel TR, Daar AS (2011). Grand challenges in global mental health. Nature.

[2] World Health Organization. Depression and other common mental disorders: World Health Organization; 2017. http://www.who.int/mental_health/management/depression/prevalence_global_health_estimates/en/. Accessed 15 May 2019.

[3] Patel V, Flisher AJ, Hetrick S, McGorry P (2007). Mental health of young people: a global public-health challenge. Lancet.

[4] Ndetei DM, Mutiso V, Maraj A, Anderson KK, Musyimi C, McKenzie K (2016). Stigmatizing attitudes toward mental illness among primary school children in Kenya. Soc Psychiatry Psychiatr Epidemiol.

[5] Patel V, Stein DJ. Common mental disorders in sub-Saharan Africa: the triad of depression, anxiety and somatization. In: Akyeampong E, Hill AG, Kleinman A, editors. The culture of mental illness and psychiatric practice in Africa. Bloomington: Indiana University Press; 2015. p. 50–72.

[6] Kilburn K, Thirumurthy H, Halpern CT, Pettifor A, Handa S (2016). Effects of a large-scale unconditional cash transfer program on mental health outcomes of young people in Kenya. J Adolesc Health.

[7] Yatham S, Sivathasan S, Yoon R, da Silva TL, Ravindran AV (2018). Depression, anxiety, and post-traumatic stress disorder among youth in low and middle income countries: a review of prevalence and treatment interventions. Asian J Psychiatr.

[8] Kumakech E, Cantor-Graae E, Maling S, Bajunirwe F (2009). Peer-group support intervention improves the psychosocial well-being of AIDS orphans: cluster randomized trial. Soc Sci Med.

[9] Ssewamala FM, Neilands TB, Waldfogel J, Ismayilova L (2012). The impact of a comprehensive microfinance intervention on depression levels of AIDS-orphaned children in Uganda. J Adolesc Health.

[10] Bolton P, Bass J, Betancourt T, Speelman L, Onyango G, Clougherty KF (2007). Interventions for depression symptoms among adolescent survivors of war and displacement in northern Uganda: a randomized controlled trial. JAMA.

[11] Shidhaye R, Kermode M (2013). Stigma and discrimination as a barrier to mental health service utilization in India. Int Health.

[12] Getanda EM, Papadopoulos C, Evans H. The mental health, quality of life and life satisfaction of internally displaced persons living in Nakuru County, Kenya. BMC Public Health. 2015;15. 10.1186/s12889-015-2085-7. 10.1186/s12889-015-2085-7 PMC4527222 26246147

[13] Osborn TL, Wasil AR, Venturo-Conerly KE, Schleider JL, Weisz JR (2020). Group intervention for adolescent anxiety and depression: outcomes of a randomized trial with adolescents in Kenya. Behav Ther.

[14] Walton GM (2014). The new science of wise psychological interventions. Curr Dir Psychol Sci.

[15] Walton GM, Wilson TD (2018). Wise interventions: psychological remedies for social and personal problems. Psychol Rev.

[16] Schleider JL, Mullarkey MC, Chacko A (2020). Harnessing wise interventions to advance the potency and reach of youth mental health services. Clin Child Fam Psychol Rev.

[17] Schleider JL, Weisz JR (2018). A single-session growth mindset intervention for adolescent anxiety and depression: 9-month outcomes of a randomized trial. J Child Psychol Psychiatry.

[18] Schleider JL, Burnette JL, Widman L, Hoyt C, Prinstein MJ. Randomized trial of a single-session growth mind-set intervention for rural adolescents’ internalizing and externalizing problems. J Clin Child Adolescent Psychol. 2020;49(5):660-72. 10.1080/15374416.2019.1622123 PMC6923626 31219698

[19] Yeager DS, Johnson R, Spitzer BJ, Trzesniewski KH, Powers J, Dweck CS (2014). The far-reaching effects of believing people can change: implicit theories of personality shape stress, health, and achievement during adolescence. J Pers Soc Psychol.

[20] Froh JJ, Kashdan TB, Ozimkowski KM, Miller N (2009). Who benefits the most from a gratitude intervention in children and adolescents? Examining positive affect as a moderator. J Posit Psychol.

[21] Cohen GL, Garcia J, Purdie-Vaughns V, Apfel N, Brzustoski P (2009). Recursive processes in self-affirmation: intervening to close the minority achievement gap. Science.

[22] Weisz JR, Kuppens S, Ng MY, Eckshtain D, Ugueto AM, Vaughn-Coaxum R (2017). What five decades of research tells us about the effects of youth psychological therapy: a multilevel meta-analysis and implications for science and practice. Am Psychol.

[23] Kenya National Bureau of Statistics (2020). Distribution of population by age, sex and administrative units. 2019 Kenya population and housing census.

[24] Ndetei DM, Khasakhala L, Nyabola L, Ongecha-Owuor F, Seedat S, Mutiso V (2008). The prevalence of anxiety and depression symptoms and syndromes in Kenyan children and adolescents. J Child Adolesc Ment Health.

[25] Osborn TL, Venturo-Conerly KE, Wasil AR, Schleider JL, Weisz JR (2020). Depression and anxiety symptoms, social support, and demographic factors among Kenyan high school students. J Child Fam Stud.

[26] Osborn TL, Wasil AR, Weisz JR, Kleinman A, Ndetei DM. Where is the global in global mental health? A call for inclusive multicultural collaboration. Gen Psychiatry. 2020;33(6):e100351. 10.1136/gpsych-2020-100351 PMC7646349 33225214

[27] Kroenke K, Spitzer RL (2002). The PHQ-9: a new depression diagnostic and severity measure. Psychiatr Ann.

[28] Spitzer RL, Kroenke K, Williams JBW, Löwe B (2006). A brief measure for assessing generalized anxiety disorder: the GAD-7. Arch Intern Med.

[29] Khasakhala L, Ndetei D, Mutiso V, Mbwayo A, Mathai M (2012). The prevalence of depressive symptoms among adolescents in Nairobi public secondary schools: association with perceived maladaptive parental behaviour. Afr J Psych.

[30] Venturo-Conerly KE, Roe E, Wasil A, Osborn TL. Training and supervising lay-providers in low-income settings: a mixed-methods study of task-sharing from the Shamiri randomized controlled trial. preprint: Open Science Framework; 2020. 10.31219/osf.io/bqznm.

[31] Schleider JL, Weisz JR (2016). Reducing risk for anxiety and depression in adolescents: effects of a single-session intervention teaching that personality can change. Behav Res Ther.

[32] Miyake A, Kost-Smith LE, Finkelstein ND, Pollock SJ, Cohen GL, Ito TA (2010). Reducing the gender achievement gap in college science: a classroom study of values affirmation. Science.

[33] Yeager DS, Dweck CS (2012). Mindsets that promote resilience: when students believe that personal characteristics can be developed. Educ Psychol.

[34] Yeager DS, Walton GM (2011). Social-psychological interventions in education: they’re not magic. Rev Educ Res.

[35] Chorpita BF, Weisz JR (2009). Modular approach to therapy for children with anxiety, depression, trauma, or conduct problems (MATCH-ADTC).

[36] Toepfer SM, Cichy K, Peters P (2012). Letters of gratitude: further evidence for author benefits. J Happiness Stud.

[37] Froh JJ, Sefick WJ, Emmons RA (2008). Counting blessings in early adolescents: an experimental study of gratitude and subjective well-being. J Sch Psychol.

[38] Kroenke K, Spitzer RL, Williams JBW, Löwe B (2010). The Patient Health Questionnaire somatic, anxiety, and depressive symptom scales: a systematic review. Gen Hosp Psychiatry.

[39] Omoro SAO, Fann JR, Weymuller EA, MacHaria IM, Yueh B (2006). Swahili translation and validation of the Patient Health Questionnaire-9 depression scale in the Kenyan head and neck cancer patient population. Int J Psychiatry Med.

[40] Osborn TL, Kleinman A, Weisz JR. Complementing standard Western measures of depression with locally co-developed instruments: a cross-cultural study on the experience of depression among the Luo in Kenya. Transcult Psychiatry. Transcultural Psychiatry: 2020. In press. 10.31234/osf.io/v3hef. 10.1177/13634615211000555 33818199

[41] Othieno CJ, Okoth RO, Peltzer K, Pengpid S, Malla LO (2014). Depression among university students in Kenya: prevalence and sociodemographic correlates. J Affect Disord.

[42] Zimet GD, Dahlem NW, Zimet SG, Farley GK (1988). The multidimensional scale of perceived social support. J Pers Assess.

[43] Weisz JR, Southam-Gerow MA, McCarty CA (2001). Control-related beliefs and depressive symptoms in clinic-referred children and adolscents: developmental differences and model specificity. J Abnorm Psychol.

[44] McCullough ME, Emmons RA, Tsang J-A (2002). The grateful disposition: a conceptual and empirical topography. J Pers Soc Psychol.

[45] Kern ML, Benson L, Steinberg EA, Steinberg L (2016). The EPOCH measure of adolescent well-being. Psychol Assess.

[46] Russell DW (1996). UCLA loneliness scale (version 3): reliability, validity, and factor structure. J Pers Assess.

[47] Schleider JL, Mullarkey MC, Weisz JR (2019). Virtual reality and web-based growth mindset interventions for adolescent depression: protocol for a three-arm randomized trial. JMIR Res Protoc.

[48] Spybrook J, Bloom H, Congdon R, Hill C, Martinez A, Raudenbush S, TO A. Optimal design plus empirical evidence: Documentation for the “Optimal Design” software. New York: William T. Grant Foundation; 2012. p. 216. Report No. 1.

[49] Buuren SV, Groothuis-Oudshoorn K. mice: Multivariate imputation by chained equations in R. J Stat Software. 2010:1–68.

[50] Nunnally JC (1978). Psychometric theory.

[51] Yara PO, Wanjohi C (2011). Performance determinants of Kenya Certificate of Secondary Education (KCSE) in mathematics of secondary schools in Nyamaiya division, Kenya. Asian Soc Sci.

[52] Mitchell S, Abbott S (1987). Gender and symptoms of depression and anxiety among Kikuyu secondary school students in Kenya. Soc Sci Med.

[53] Osborn TL, Rodriguez M, Wasil AR, Venturo-Conerly KE, Gan J, Alemu RG (2020). Single-session digital intervention for adolescent depression, anxiety, and well-being: outcomes of a randomized controlled trial with Kenyan adolescents. J Consult Clin Psychol.

