# Supplementary material for: The Shamiri group intervention for adolescent anxiety and depression: study protocol for a randomized controlled trial of a lay-provider-delivered, school-based intervention in Kenya
Source: Trials. 2020 Nov 23;21:938. doi: 10.1186/s13063-020-04732-1 (PMC7682107; doi:10.1186/s13063-020-04732-1)
Supplement: Supplementary file 1 — Additional file 1. [file 13063_2020_4732_MOESM1_ESM.docx]

Hatujambo!

Today, we are going to talk about something called “neuroplasticity.” Neuroplasticity is the fact that your brain can grow if you want it to; you can always learn new skills and improve your personality. Brain scientists from universities around the world including Harvard University and Stanford University have been studying how the brain grows and changes for many years now. They have learned that the brain doesn’t stop growing when you are a small child. In fact, our brains can grow during our whole life! Now, of course, the fact that our brains can grow doesn’t mean that the shape of our heads will start changing or anything like that. It means that throughout our lives, our brains have “neuroplasticity”, or the ability to form new ways of working. When our brains learn these new ways of working, our personalities and skills can change.

To understand how neuroplasticity works, imagine the brain as having different roads inside it that it uses when you think, feel, or do different things. Some of these roads are used often. For example, the roads for walking, talking, doing math, and speaking Swahili are very well traveled and common roads, because we do these activities often. Through repetition and practice, it becomes easier and easier for out brain to travel these roads. So, when we want to teach ourselves a new habit or learn to do something different, the more we practice it, the easier it becomes for our brain to do it. As we do something more often, the road for it in our brain becomes easier to use. This is neuroplasticity, and all of us can use neuroplasticity to help us grow to become better.

Neuroplasticity means that we can teach ourselves to think of challenges differently. Often, as we are growing and learning new things, we will make mistakes along the way. But neuroplasticity shows us that these mistakes are just challenges on the path to growth; We can, through effort and strategies, improve over time as our brain learns new ways of working. We might sometimes feel sad about making a mistake, but now we know that if we work hard and learn new habits, we can eventually grow and get better.

Let’s say I take an exam and get a really bad score. Instead of being sad and thinking I’m not smart, I try to think of what I can do differently before my next exam, because I know that I can improve if I try. If there is something I don’t understand, maybe I should pay more attention in class, ask a friend for help, or ask questions in class. And I will do all these things and wait to see what my new score will be!

With a growth mindset, I will also start seeing mistakes as opportunities to learn more. Because they will help me find out something I’m doing wrong, so, thanks to my mistake, I can learn how to do things better the next time! This is what growth mindset is all about!
